# Development and Application of Microsatellites in *Carcinus maenas*: Genetic Differentiation between Northern and Central Portuguese Populations

**DOI:** 10.1371/journal.pone.0007268

**Published:** 2009-09-30

**Authors:** Sónia Pascoal, Simon Creer, Martin I. Taylor, Henrique Queiroga, Gary Carvalho, Sónia Mendo

**Affiliations:** 1 CESAM & Departamento de Biologia, Universidade de Aveiro, Campus Universitário de Santiago, Aveiro, Portugal; 2 School of Biological Sciences, College of Natural Sciences, Environment Centre Wales, Bangor University, Gwynedd, United Kingdom; Erasmus University Medical Center Rotterdam, Netherlands

## Abstract

*Carcinus maenas*, the common shore crab of European coastal waters, has recently gained notoriety due to its globally invasive nature associated with drastic ecological and economic effects. The native ubiquity and worldwide importance of *C. maenas* has resulted in it becoming one of the best-studied estuarine crustacean species globally. Accordingly, there is significant interest in investigating the population genetic structure of this broadly distributed crab along European and invaded coastlines. Here, we developed polymerase chain reaction (PCR) primers for one dinucleotide and two trinucleotide microsatellite loci, resulting from an enrichment process based on Portuguese populations. Combining these three new markers with six existing markers, we examined levels of genetic diversity and population structure of *C. maenas* in two coastal regions from Northern and Central Portugal. Genotypes showed that locus polymorphism ranged from 10 to 42 alleles (N = 135) and observed heterozygosity per locus ranged from 0.745 to 0.987 with expected heterozygosity ranging from 0.711 to 0.960; values typical of marine decapods. The markers revealed weak, but significant structuring among populations (global F_ST_ = 0.004) across a 450 km (over-water distance) spatial scale. Combinations of these and existing markers will be useful for studying population genetic parameters at a range of spatial scales of *C. maenas* throughout its expanding species range.

## Introduction

*Carcinus maenas* (Linnaeus 1758) is the most common crab species of European estuarine waters with a native distribution extending along the Atlantic coasts of Europe and Northern Africa, from Norway to Mauritania [1]. In Portuguese coastal waters, *C. maenas* is one of the most prevalent crab species and is of high ecological and commercial value. In local estuaries, *C. maenas* is collected at the intermoult stage and is used in large quantities (hundreds of tons per year) by the Iberic food industry as scent for fish-paste based products. *Carcinus* is also sold as valuable bait for anglers in several countries, meaning that the harvest and sale of *C. maenas* products is wide-ranging and is estimated at millions of Euros.

The species is an extremely successful predator [2], [3] and is highly tolerant to a broad range of environmental conditions [4], [5]. Consequently, the species' ecological characteristics have facilitated a globally invasive expansion during the last century, establishing populations in many parts of the world [6]–[10]. In some areas its range continues to expand, with measurable impacts on native communities [11]. *C. maenas* is currently included in the World Conservation Union (IUCN) list of the 100 most dangerous invasive species [12]. It is therefore important to understand the scale and dynamics of dispersal and gene flow in this crustacean, information that, until recently [13], has been constrained by the availability of sufficiently informative genetic markers.

**Figure 1:**
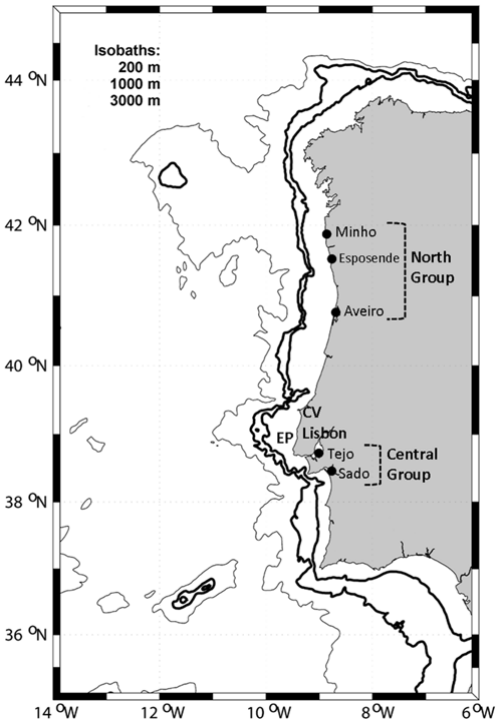
Map showing the west coast of the Iberian Peninsula and sampling site location. CV: Cape Carvoeiro; EP: Estremadura Promontory.

*C. maenas* has high fecundity [14] and a long planktonic larval phase [15], [16]. Moreover, previous studies indicate that larval release at nocturnal high tide and inherited vertical-migration rhythms further enhance exportation from estuaries and rocky shores [17] into offshore hydrodynamic currents. Offshore dispersal may therefore account for considerable exchange of individuals among local populations [18], and according to population genetics theory, marine taxa with planktonic larvae are estimated to possess lower population genetic structuring, with higher connectivity than those with direct development [19]. Dispersal of larvae depends to a large degree on the temporal variability of the current patterns, which are strongly affected by winds, density stratification of the water column and instabilities. Dispersal also depends on larval behaviour and on its capacity to regulate vertical position in the water-column [20]. Research during the last two decades has highlighted the importance of the dispersal ability of planktonic larvae of marine organisms in the spread, establishment and maintenance of populations (e.g. [21]–[24]). In Portugal, the Estremadura Promontory (see Figure 1) and other significant topographic structures such as canyons and river runoff cause differences in the shelf oceanography of the Western Iberian basin [25]. South of the Estremadura Promontory, the continental shelf is narrow and steeply sloping, but to the north, the shelf is wider and less steep. River runoff is significant to the north of Lisbon where several rivers contribute fresh water input to the shelf [25]. Additionally, several submarine canyons exist to the north and to the south, which may affect local surface circulation. In the context of population connectivity, it is proposed that proximate estuaries and rias of western Iberia are inter-linked populations sharing similar conditions of dispersal and retention, including coastline orientation, alternating winds during late winter and spring, and river plumes. Such conditions are notably different between North and South of the Cape Carvoeiro (see Figure 1) [25]. Accordingly, sampling regimes for population genetic studies in the Portuguese/Iberian coasts should take such topographic and hydrodynamic data into consideration as they may significantly influence larval dispersal and associated recruitment dynamics.

A study analysing the mitochondrial (mtDNA) cytochrome *c* oxidase I (COI) gene from crabs collected in European populations of *C. maenas* [19] detected a clear break between populations of the Mediterranean and Atlantic, supporting the species-level status between *C. maenas* and *C. aestuarii*, as well as significant differentiation among populations from Iceland and the Faeroe islands and those from continental Europe. The study ascribed the subdivisions to isolation by distance along European coastlines, and to a deep-water barrier between the continental shelf and off-shore islands. In the latter case it is unclear how a deep-water basin should constitute a barrier to larvae that dwell in surface waters, and an alternative explanation could be local selection on functional genes caused, for instance, by lower temperatures in the off-shore islands. Despite the discovery of significant phylogeographic structuring at larger spatial scales, mitochondrial DNA variation along the coast of continental Europe was slight [19]. In order to complement and extend previous work,, characterization of fine-scale population structure analyses based on several unlinked nuclear loci is desirable. The first set of 14 microsatellite DNA markers for *C. maenas* was published by Tepolt et al. [26] followed very recently by an additional marker published in Darling et al. [13]. Following initial trials of these available markers, excluding the latter marker, unpublished at the time of this work, it became clear that not all loci co-amplify populations from disparate genetic ranges. Accordingly, we embarked on a further round of microsatellite marker enrichment in order to have an appropriately robust suite of informative population genetic markers for *C. maenas*.

The main objectives of the present study were to i) develop new molecular markers for *C. maenas* ii) study the genetic differentiation and population structure for the green crab at selected spatial scales within its native range and iii) explore the relationships between genetic differentiation and hydrodynamic/topographic barriers at regional scales. Therefore, here we describe three new polymorphic microsatellite loci sourced from Portuguese populations, together with analyses of a further six pre-existing loci, to undertake an initial hierarchical analysis of genetic diversity among *C. maenas* along the Portuguese coast.

## Materials and Methods

### Sampling

Given the potential hydrodynamic differentiation between North and South of the Cape Carvoeiro [25], two sampling regions representative of northern and central populations were chosen for the present study. For the North Coast group, populations were collected from three estuaries (Minho, Esposende and Aveiro). Two further estuaries (Tejo and Sado) were pooled to form the Central Coast group (Figure 1 and Table 1). These two regions are separated by approximately 250 km (over-water distance) and by the Estremadura Promontory (39°N).Sampling (N = 135) was performed from April to July 2005 using baited nets. Crab pereiopods were removed and stored at −80°C until used for DNA extraction.

**Table 1:** Sampling sites details.

	Latitude	Longitude	Sample size
**Minho**	41°52′N	8°50′W	29
**Esposende**	41°31′N	8°46′W	18
**Aveiro**	40°37′N	8°44′W	30
**Tejo**	38°44′N	8°55′W	25
**Sado**	38°24′N	8°45′W	33

### Enriched genomic libraries

The isolation and characterization of the microsatellite loci were conducted with slight modifications for enriched genomic libraries, described in Zane et al. [27]. Crabs used for developing microsatellites were collected from three populations along the Portuguese Coast: Esposende, Aveiro and Sado (Figure 1). Total genomic DNA was extracted from muscle tissue in periopods of six different *C. maenas* (two from each population) using a DNeasy™ Tissue Kit (Qiagen) and then pooled into a single volume. After digestion of DNA with the restriction enzyme *Sau*3A (Fermentas), fragments were ligated to specific adaptors: SAULA 3′-GGTTCGAAGGGCCCATGGCG-5′ and SAULB 5′-GATCCCAAGCTTCCCGGGTACCGC-3′. The DNA ligated to the adaptor was PCR-amplified using the 20-mer oligonucleotide SAULA adaptor as the primer. The adaptor-ligated DNA fragments were selected by hybridization to biotinylated oligonucleotides [(CA)_12_, (GA)_12_, (CAG)_8_] and captured with streptavidin-conjugated magnetic beads (Dynabeads, DYNAL). Hybridizing DNA fragments were eluted and PCR-amplified using the 20-mer oligonucleotide SAULA adaptor as the primer. These amplicons were subjected to a second round of hybridization and PCR amplification. The adaptors were cleaved internally with *Mbo*I (Fermentas), after which the microsatellite-enriched library was cloned into a pUC19 vector (Promega) and transformed into competent *Escherichia coli* DH5α cells. Clones containing microsatellites were identified by dot blot hybridisation with biotinylated oligonucleotides [(CA)_12_, (GA)_12_, (CAG)_8_]. Cloned inserts were amplified and sequenced using the Macrogen™ (www.macrogen.com) sequencing facility, using M13 universal primers for both strands. Primers were designed using PRIMER 3 software [28] for sequences with the appropriate repetitive elements and sufficiently extensive flanking regions to allow primer development. Various permutations of PCR conditions were tested on all primer pairs to optimize locus-specific amplification conditions and test their utility as genetic markers.

PCR amplifications were carried out in 15 µl reactions containing 10–100 ng of genomic DNA, 0.3 pmol of each primer, 1× PCR buffer, 1.5 mM MgCl_2_, 0.2 mM of each dNTP (Promega) and 0.5 U Taq DNA polymerase (Promega) on an MJ Mini Thermal-Cycler (Biorad). Thermal cycling parameters were: initial denaturation at 94°C for 5 min followed by 35 cycles of denaturation at 94°C for 1 min, primer-specific annealing temperature for 1 min (Table 2), extension at 72°C for 1 min and final extension at 72°C for 10 min. Microsatellite fragments were then resolved on 1.5% agarose gels. Some loci were selected for further investigation, and in those cases forward primers were 5′ labelled with a fluorescent dye (HEX or 6-FAM) to generate fluorescently-labelled microsatellite fragments.

**Table 2:** Main genetic variability measures by locus of *Carcinus maenas* from the Portuguese coast.

Locus	Primer sequence 5′-3′	Repeat	T (°C)	Size range (bp)	Na	H_E_	H_O_	F_IS_	F_ST_	H-W
**Cma03EPA**	F:CGCTCGACATGCTGTATTGT	(TAGA)_16_	57	130–202	17	0.850	0.987	−0.159	−0.002	1.000
	R:CAATTTATCTATCCATCTCTATCCTTC									
**Cma04EPA**	F:GAGCTCCAGGAAACTGTATCTGA	(TAGA)_10_	57	131–223	17	0.885	0.959	−0.077	0.001	0.424
	F:GCCCTCTATCTCGCTTTATATCTC									
**Cma07EPA**	F:TCAGGGCCAAAAGTTATTCAA	(TAA)_21_	59	136–199	20	0.930	0.981	−0.049	0.007	0.961
	R:GTTGTTGGCATTCGCTCTTT									
**Cma10EPA**	F:GAGACCGTCAATGCAGCTTCCTCT	(CA)_37_	59	226–302	36	0.958	0.877	0.088	0.006	0.006
	R:GGGACAGAACGTATCTAGGTCACC									
**Cma11EPA** **	F:AGTAGGCGTCCTTTGTTTCAG	(CA)_53_	55	174–276	42	0.960	0.765	0.209	0.002	P<0.001
	R:CGTTGATTTGATGTTACTTTTAGG									
**Cma14EPA**	F:ACGGCTCACCTACGTGCACT	(CCA)_8_	60	216–267	10	0.558	0.649	−0.173	0.009	0.998
	R:GGCTGTGGTCCTGTGTTCATT									
**SP107** *	F:GTACCCGGGAAGCAGAGAAC	(GAG)_16_	51	150–189	14	0.716	0.864	−0.196	0.010	1.000
	R:CACTTGCTATAAAGGCCTCAGC									
**SP251** *	F:TGGTACTGTGCGTGGTGAAGC	(CA)_38_	59	201–269	32	0.948	0.760	0.194	0.005	P<0.001
	R:TGTGGTACGATGCGGCATAG									
**SP495** *	F:AAGTTCCAGGGCCTGAGTGTA	(CAG)_10_	52	142–193	15	0.711	0.745	−0.045	−0.004	0.422
	R:TAGTGGTGGTGGTGGTGGAAT									
**All**					203	0.835	0.843	−0.007	0.004	

T (°C): annealing temperature; bp: base pairs; Na: number of alleles found per locus; H_E_: expected heterozygosity; H_O_: observed heterozygosity; F_IS_: standardised genetic variance within populations at each locus; F_ST_: standardized genetic variance among populations at each locus; H-W: Hardy-Weinberg P values.

* Microsatellites developed in the present study.

** Minor adjustments to Tepolt et al. [30] F: AGGCGTCCTTTGTTTCAGTT ; R:TTGATTTGATGTTACTTTTAGGATGT.

In order to determine levels of polymorphism of these new putative markers, we screened 18 individual samples collected from the Sado estuary. Extension products were resolved on an ABI PRISM 310 Genetic Analyser (Applied Biosystems) and alleles were sized relative to an internal size standard (ROX GS 400HD; Applied Biosystems) using the GENESCAN 3.7 software (Applied Biosystems).

Additionally, in order to identify a robust suite of reliable markers, all 14 microsatellites described by Tepolt et al. [26] were tested with the same group of crabs and, of these, the six markers that exhibited the most consistent and robust amplification were used for further analyses. The microsatellite described by Darling et al. [13] was not tested in the present study as it was published after the empirical work described here.

### DNA extraction and microsatellite analysis

Total genomic DNA was extracted from muscle tissue in periopods of all *C. maenas* using the DNeasy Tissue Kit (Qiagen). Following initial trials, we tested six microsatellite loci described by Tepolt et al. [26]: Cma03EPA (GenBank DQ131484), Cma04EPA (GenBank DQ131485), Cma07EPA (GenBank DQ 131488), Cma10EPA (GenBank DQ131491), Cma14EPA (GenBank DQ131495) and Cma11EPA (GenBank DQ131492) with slight modifications (Table 2). We also tested the three newly developed primer pairs SP251 (GenBank EU378909), SP107 (GenBank EU378911) and SP495 (GenBank EU378914) for the 135 crabs. PCR amplifications were carried out in 15 µl reactions with 10 pmol of each primer set (forward primer in each pair was 5′ labelled with a fluorescent dye), 10–100 ng of genomic DNA, 1× PCR buffer, 1.5 mM MgCl_2_, 0.2 mM of each dNTP (Promega) and 0.5 U Taq DNA polymerase (Promega) on a MJ Mini Thermal-Cycler (Biorad). Thermal cycling parameters were: initial denaturation at 94°C for 5 min followed by 35 cycles of denaturation at 94°C for 1 min, primer-specific annealing temperature (Table 2) for 1 min, extension at 72°C for 1 min and final extension at 72°C for 10 min. Following PCR amplification, the extension products were resolved on 1.5% agarose gels. Genotyping was performed on an ABI PRISM 310 Genetic analyser (Applied Biosystems) and alleles were sized relative to an internal size standard (ROX GS 400HD; Applied Biosystems) using GENESCAN 3.7 (Applied Biosystems).

### Statistical Analysis

Initially the raw data was analysed with MICRO-CHECKER [29] to check microsatellites for null alleles and scoring errors. Excel Microsatellite Toolkit [30] was used to calculate allelic frequencies, mean number of alleles per locus and observed (H_0_) and expected heterozygosity (H_E_) under Hardy-Weinberg assumptions. Tests for deviations from Hardy-Weinberg proportions, heterozygote deficiencies, genotypic linkage equilibrium and genic heterogeneity among populations were estimated using the exact test of GENEPOP version 3.4 [31]. Estimates of F_ST_, F_IS_, and their significance per population over all loci were calculated using FSTAT version 2.9.3.2 [32]. Finally, hierarchical analysis of molecular variance (AMOVA) was performed using GenAlEx version 6.2 [33] in order to test for possible regional structure.

## Results

### Enriched genomic libraries

Screening of 144 positive clones from the enriched genomic library yielded a total of 48 unique microsatellite sequences, though only twenty-four of these showed sufficiently extensive flanking regions to allow primer development. Out of the 24 primer pairs tested, seven failed to produce amplification products under any of the conditions tested. To confirm that the primers were not useful, each pair was subjected to a round of PCR optimization performing cycling with annealing temperatures ranging from 45°C to 62°C and MgCl_2_ concentrations ranging from 1 mM to 4 mM. Under the same test conditions, eleven other loci were either monomorphic or were unusable because of spurious peaks and/or extensive and stuttering. The remaining six microsatellite loci tested were polymorphic but only three could be amplified reliably (Table 2). Preliminary genotyping of individuals showed that locus polymorphism ranged from 7 to 21 alleles (N = 18) and observed heterozygosity (H_O_) per locus ranged from 0.67 to 0.94 with the expected heterozygosity (H_E_) from 0.67 to 0.97. No evidence of linkage disequilibrium was observed, and a test for concordance with Hardy-Weinberg equilibrium (HWE) revealed deviation from HWE in locus SP251. The primers described by Tepolt et al. [26] yielded generally uniform amplifications of putative loci in the Sado population, though locus Cma11EPA in the present study has minor adjustments (Table 2). Some primer pairs (Cma06EPA Cma09EPA and Cma12EPA) exhibited inconsistencies in amplification and were difficult to genotype in our samples due to excessive stuttering, and the Cma13EPA primers failed to amplify the putative locus in any individuals, even after testing a range of temperatures and MgCl_2_ concentrations.

### Microsatellite analysis

A total of 135 *C. maenas* retrieved from the two geographic regions were typed for the nine microsatellite loci. The Micro-checker analysis did not detect scoring errors. However, null alleles may be present in locus Cma10EPA, Cma11EPA and SP251 due to an excess of homozygotes, and corresponding significant positive values of F_IS_ (Table 2). All nine microsatellite loci were polymorphic for the studied populations and the number of alleles per population per locus ranged from 9 to 35, with a total number of 203 alleles in the global sample. Levels of genetic variability were similar across samples. The expected heterozygosity (H_E_) per locus ranged from 0.711 to 0.960, and the observed heterozygosity (H_O_) from 0.745 to 0.987 (Table 2). All individual loci, except those with apparent null alleles, show higher observed than expected heterozygosity. The expected heterozygosity (H_E_) across all loci per population was 0.843 and 0.827, and the observed heterozygosity (H_O_) was 0.838 and 0.848 in the North and Central Group respectively (Table 3).

**Table 3:** Main genetic variability measures for Portuguese coast *Carcinus maenas* populations.

Group of populations	N	H_exp_ (±SD)	H_obs_ (±SD)	N-all (±SD)	F_IS_									
					Cma10	Cma11	Cma14	SP251	SP495	SP107	Cma03	Cma04	Cma07	All
**North Group**	77	0.8430±0,04	0.8384±0,01	19.89±9.79	0,105	0,242	−0,232	0,173	−0,031	−0,135	−0,142	−0,054	−0,025	0.006
**Central Group**	58	0.8274±0,05	0.8476±0,02	19.22±9.19	0,065	0,164	−0,076	0,222	−0,065	−0,283	−0,181	−0,113	−0,084	−0.025

N: sample size; H_exp_: unbiased heterozygosity according to Hardy-Weinberg; H_obs_: observed heterozygosity; N-all: mean number of alleles per locus and standardised genetic variance within populations (F_IS_) at each locus for each population; SD: Standard deviation.

A global test for concordance with HWE revealed deviations from HWE in locus Cma10EPA, Cma11EPA and SP251 (Table 2). Testing HWE for individual populations and loci revealed that this disequilibrium remained significant within populations (all populations at locus Cma11EPA, Tejo, Sado and Minho populations for locus SP251 and Tejo and Aveiro populations for locus Cma10EPA). Evidence of linkage disequilibrium was observed in pairwise loci SP251/SP495, SP251/Cma14EPA and Cma14EPA/SP495 in the global population test. Global F_IS_ value was −0.007, suggesting an excess of heterozygotes in the sampling area. F_ST_ values per locus ranged from −0.004 and 0.010, and the global F_ST_ was 0.004 (P<0.001) revealing weak but significant structuring.

Additionally, hierarchical AMOVA (Table 4) revealed that most of the genetic variance was found within populations (92%, P<0.001); however, a significant fraction (7%) was also found to partition among biogeographic regions (North Coast Group and Central Coast Group) and among populations within regions (1%).

**Table 4:** Hierarchical AMOVA for *Carcinus maenas* populations in the Portuguese coast.

Source of variation	d.f.	SS	MS	Est. Var.	(% )	P = value
**Among regions**	1	43.036	43.036	0.511	7	P<0.001
**Among populations within regions**	3	27.178	9.059	0.087	1	0.001
**Within populations**	130	880.638	6.774	6.774	92	P<0.001
**Total**	134	950.852	58.87	7.372		

d.f.: degrees of freedom; SS: sum of squares; MS: mean squares; Est. Var.: Variance component; (%): percentage of total variation contributed by each component and its associated significance (P = value).

## Discussion

Here, we developed three new highly polymorphic microsatellite loci for *C. maenas*, chosen from two different dinucleotide (CA and GA) and one trinucleotide (CAG) enriched genomic libraries. Despite the many positive clones derived from the enriched genomic libraries, we experienced difficulties in developing new microsatellite loci: short sequences, no flanking regions in one or in both ends, PCR instability, stutter and difficult genotyping all contributed to a reduced number of employed markers. Other researchers have also experienced difficulties in isolating microsatellite loci from marine invertebrate species, mainly because microsatellite repeats in invertebrates are typically less abundant and shorter than in vertebrates [34].

The microsatellites generally exhibited high levels of heterozygosity and, except for loci Cma10EPA, Cma11EPA and SP251, observed heterozygosity was not-significantly higher than expected heterozygosity. Significant deviations from Hardy-Weinberg equilibrium between and within populations (exact probability test, P<0.05) were recorded for loci Cma10EPA, Cma11EPA and SP251, and are most likely due to the presence of null alleles and an excess of homozygotes at these loci.

Our study showed a global F_ST_ of 0.004 (P<0.001) between the Northern and Central Groups, suggesting weak, but significant structuring, among the sampled populations collected in 2005. Accordingly, significant regional genetic structure between these two biogeographic regions was also revealed by AMOVA. The genetic differentiation may be the result of neutral population differentiation, or the effects of selection acting on functional genes correlated with the neutral markers [35], [36]. There is a persistent, although weak, gradient in temperature along the West Iberian coast, with yearly-averaged sea surface temperature in the North coast of Portugal 2–3°C lower than in the Central coast [37]. However, given that *C. maenas* is continuously distributed over a considerably larger range of temperatures and of other environmental conditions with no signature of a genetic break such as observed along the coast of continental Europe [13], and in view of the weak level of structuring, we suggest that neutral population differentiation is a more likely explanation of the observed differences.

*C. maenas* has a long planktonic larval phase (4 to 6 weeks [17]). A previous study using a high resolution nested numerical oceanographic model, coupled with individual-based models to simulate vertical migration behaviour, suggested that the dispersal radius during planktonic dispersal for this species is within 200 km (120 km on average) along the west coast of the Iberian Peninsula [25]. The estimate is consistent with those based on the advancement of population boundaries in coasts recently invaded by *C. maenas* [38] and on empirical observations on the strength of coastal currents. Therefore, because most estuarine populations along the Portuguese coast are mainly separated by distances of 20 to 60 km, such a dispersal radius is predicted to result in considerable exchange of individuals among local populations. However, our sampling areas are separated by approximately 250 km (shortest over water distance) and, apart from small and transient populations associated with non-permanent estuaries and coastal lagoons, are potentially linked by just one additional estuarine habitat - Figueira da Foz (194 km from Tejo and 59 km from Aveiro). According to the oceanographic model [25], the distance between this estuary and the Central Group is greater than the predicted megalopa average dispersion radius, potentially contributing to the genetic differentiation observed here.

Recent information [13] regarding patterns of genetic diversity of *C. maenas* throughout its global expansive range revealed significant partitioning of genetic variance between Western and Northern Europe populations by both microsatellite and mitochondrial data. However, weaker evidence of native structure was observed using microsatellite data, compared to mtDNA COI analysis. In the former case, only comparisons between off-shelf populations of Iceland and Feröe Islands, with continental native populations showed consistent differences. Darling et al. [13] explained the discrepancy between marker sets by either the greater sensitivity of mitochondrial loci to the effects of genetic drift, or by homoplasy in the microsatellite data.

Previous studies on population genetics of marine organisms along the Portuguese coast analysed one seagrass (*Zostera noltii* [39]), two crabs (*Necora puber* [40] and *Maja brachydactyla* [41] and one mussel (*Mytilus galoprovinciallis* [42]). Interestingly, only the seagrass exhibited strongly significant differentiation between populations located to the North and to the South of the Estremadura Promontory, indicating that most invertebrates with larval development times over 2–3 weeks show at best a weak structuring in this region. Nevertheless, the present study suggests that, despite the potential for high connectivity between local populations of invertebrate species with planktotrophic larval development within the Portuguese margins, some local constraints to gene flow among populations of *C. maenas* may exist, at least during the time period in which sampling was undertaken. Such constraints might result from hydrodynamic or topographic barriers along the studied area, with a particular impact on dispersal of the Estremadura Promontory at local levels.

Our study raises interesting questions regarding the effect of a temporally and spatially dynamic hydrogeographic regime along the Portuguese coastline. However, future studies employing additional hierarchical sampling at larger geographic scales would be desirable to complement our findings and inferences on the potential role of hydrodynamic/topographic barriers. Based on our data, and experience with a range of *C. maenas* microsatellite markers derived from geographically disparate populations, we anticipate that the combination of the current loci together with those of Tepolt et al. [26] and Darling et al. [13] will facilitate the application of an appropriately robust panel of informative polymorphic markers for population genetic studies. Together, they will be useful in examining population differentiation at a range of spatial and temporal scales in this important globally invasive species.
